# Determinants of Overweight or Obesity among Men Aged 20–59 Years: A Case-Control Study Based on the 2016 Ethiopian Demographic and Health Survey

**DOI:** 10.1155/2021/6627328

**Published:** 2021-04-23

**Authors:** Yohannes Tekalegn

**Affiliations:** Madda Walabu University, Goba Referral Hospital, School of Health Science, Department of Public Health, Goba, Ethiopia

## Abstract

**Background:**

Evidence shows that overweight or obesity has become a major public health problem in both developed and developing countries. However, there are limited studies conducted to identify the risk factors of overweight or obesity in Ethiopia. Therefore, this study aimed to assess the determinants of overweight or obesity among men aged 20–59 years in Ethiopia.

**Methods:**

This study used the 2016 Ethiopian Demographic and Health Survey (EDHS) data. A case-control study was conducted based on the EDHS data; cases were men who were overweight or obese, depending on their body mass index, and controls were men with normal body mass index. Bivariate and multivariate binary logistic regression was performed to assess the determinants of overweight or obesity among the study participants.

**Results:**

A total of 610 cases and 2440 controls were included in this study. Men aged 30–39 years (adjusted odds ratio (AOR) = 2.2, 95% CI: 1.6–3.0) and ≥40 years (AOR = 3.4, 95% CI: 2.5–4.7) had higher odds of being overweight or obese compared to men aged 20–29 years old. The likelihood of overweight or obesity was significantly higher among married men (AOR = 1.5, 95% CI: 1.1–2.0), living in urban areas (AOR = 3.1, 95% CI: 2.1–4.4), those in the rich wealth quintile (AOR = 1.9, 95% CI: 1.2–2.9), and those with primary (AOR = 1.6, 95% CI: 1.1–2.3), secondary (AOR = 2.6, 95% CI: 1.7–3.9), and higher education (AOR = 3.6, 95% CI: 2.4–5.6). Additionally, men watching television at least once a week had higher odds (AOR = 1.5, 95% CI: 1.1–2.1) of being overweight or obese.

**Conclusion:**

Men in the higher wealth quintile, older age, married, higher educational status, watching television at least once a week, urban dwellers, residents of big cities such as Addis Ababa and Harari, and residents of low land like Afar were more likely to be overweight or obese. Therefore, it is essential to design strategies and programs to reduce or prevent overweight or obesity with a special focus on the identified risk factors.

## 1. Introduction

The World Health Organization (WHO) has defined obesity as a condition with excessive fat accumulation in the body, to the extent that health is adversely affected [1]. Overweight or obesity results from a positive energy imbalance expressed by a body mass index (BMI) of 25–29.9 and ≥ 30 kg/m^2^, respectively [2, 3]. Also, in 2000, the WHO labeled obesity as the most blatantly visible, but most neglected, public health problem worldwide [4]. Overweight or obesity has become a major public health problem in both developing and developed countries as they are causally related to a wide spectrum of chronic noncommunicable diseases including type 2 diabetes, cardiovascular diseases, and cancer [5]. Both generalized obesity and abdominal obesity are associated with an increased risk of morbidity and mortality [6]. Moreover, several studies reported obesity as the well-documented major risk factor for many noncommunicable diseases and health conditions including hypertension, high lipid concentrations, type-2 diabetes, coronary heart disease, stroke, and certain cancers [5, 7–11]. According to the WHO report, in 2016, more than 1.9 billion adults aged 18 years and older were overweight or obese [12]. The global burden of overweight or obesity is recorded to be 4 million deaths and 40 million disability-adjusted life years among adults globally in 2015 [13].

The prevalence of overweight or obesity is increasing globally in both developing and developed countries, especially, those with rapid cultural and social changes [14–19]. Many low- and middle-income countries are now facing a “double burden” of disease. While these countries continue to deal with the problems of infectious diseases and undernutrition, they are also experiencing a rapid increase in noncommunicable disease risk factors such as obesity and overweight, particularly in urban settings [12].

In Africa, the body mass index (BMI) increased over time across all regions, which parallels the global average. The prevalence of overweight or obesity is estimated to be 20%–50% by 2025 in Africa [20]. In Sub-Saharan Africa countries, overweight/obesity levels are still lower than in high-income countries but certainly higher than they were two decades ago and increasing at alarming rates [21, 22]. Demographic Health Survey (DHS) analysis of 32 Sub-Saharan African countries revealed that the pooled prevalence of overweight in the region was 15.9% with the lowest in Madagascar (5.6%) and the highest in Swaziland (27.7%); similarly, the prevalence of obesity was also the lowest in Madagascar (1.1%) and the highest in Swaziland (23.0%) [23].

In Ethiopia, noncommunicable diseases (NCDs) account for 42% of deaths. Among these, 27% are premature deaths occurring before 70 years of age. Disability-adjusted life years (DALYs) due to NCDs in the country have increased from 20% in 1990 to 69% in 2015, which is more than double that of communicable maternal, neonatal, and nutritional problems combined [24, 25]. The analysis of the Ethiopian Demographic Health Survey (EDHS) found that the prevalence of overweight or obesity in urban settings was 12.1% and 2.8%, respectively [26]. Recently, the prevalence of adult overweight or obesity has increased from 4% in 2000 to 6% in 2016 [27]. Similarly, different pocket area studies showed that the prevalence of adult overweight ranges from 16.1% to 25.3% and obesity ranges from 5.6% to 16.2% [26, 28–30]. As it is known, overweight or obesity has become a complex problem resulting from a combination of genetic, behavioral, cultural, and environmental influences [31–33].

Although many studies reporting the prevalence and determinants of overweight or obesity, there are limited studies on this aspect that reported determinants of overweight or obesity among men aged 20–59 years at the national level. Therefore, identifying determinants will have paramount importance in the prevention and control of these emerging public challenges in Ethiopia. The information can be used as baseline evidence for program planners, policymakers, and researchers working on the prevention of chronic noncommunicable diseases. It will also help community members by providing information on the risk factors of overweight or obesity.

## 2. Methods and Materials

### 2.1. Data Sources

This case-control study was conducted using 2016 Ethiopian Demographic and Health Survey (EDHS), the most recent national survey conducted by the Ethiopian Central Statistical Agency (ECSA). Data collection took place from January 18, 2016, to June 27, 2016 [34].

### 2.2. Sampling Procedures and Population

The 2016 EDHS data was collected using a sampling frame from the Ethiopian population and housing census which was conducted in 2007 by the Ethiopian Central Statistical Agency. The survey was designed to represent the country as a whole, for urban and rural areas separately. All of the nine administrative regions and the two city administrations in Ethiopia were included in the survey. A two-stage stratified cluster sampling was used. Each region and one city administration were stratified into urban and rural, except Addis Ababa, which is entirely urban. A total of 645 enumeration areas (202 in urban areas and 443 in rural areas) were selected with probability proportional to the size of enumeration areas and with independent selection in each sampling stratum. In the second stage, a fixed number of 28 households per cluster were selected with an equal probability systematic selection from the newly created household listing [34]. To calculate the nutritional status of men, we merged anthropometric variables from household members recode (PR file) to the men's recode (MR file) using the cluster, household, and line numbers [35]. After merging both datasets, there were a total of 610 overweight or obese samples, based on their body mass index (BMI), and all of them were included in the analysis as a case. We used one to four cases to control the ratio; thus, 2440 samples were randomly selected from a total of 7059 men with normal BMI.

### 2.3. Data Collection and Anthropometric Measurements

EDHS survey data was collected using a structured interviewer-administered questionnaire. All men aged 20–59 years who were either permanent residents of selected households or visitors who stayed in the household the night before the survey were eligible to be interviewed for a man's questionnaire. The interview was carried out using tablet computers to record responses. Height and weight of all men aged 20–59 were measured. Weight was measured using lightweight SECA mother-infant scales with a digital screen designed and manufactured under the guidance of UNICEF. Height measurements were carried out using a Shorr measuring board. A detailed explanation of sampling procedures and the data collection process can be found elsewhere [34].

### 2.4. Outcome Variable

Overweight or obesity is the outcome variable of this study which was derived from the body mass index (BMI) data. BMI was calculated as weight in kilogram divided by the square of height in meters. Men with BMI greater than or equal to 25.0 kg/m^2^ were categorized as overweight or obese and characterized as cases. Men with a BMI of 18.5–24.99 kg/m^2^ were categorized as normal-weight men and considered as controls for this study [6].

### 2.5. Independent Variables

This study included demographic, economic, and geographic variables to assess determinants of overweight or obesity among men aged 20–59 years. Depending on the availability of the data, biological plausibility, and evidence from previously published literature, the following variables were included as explanatory variables: age (which is categorized as 20–29, 30–39, and ≥ 40 years old), marital status (recoded as single, married, and separated/divorced/widowed), educational status (recoded as no education, primary, secondary, and higher education), current employment status (working and not working), frequency of watching television (not at all, less than once a week, and at least once a week), frequency of listening to the radio (not at all, less than once a week, and at least once a week), frequency of reading newspaper or magazine (not at all, less than once a week, and at least once a week), wealth index (recoded as poor, middle, and rich), place of residence (urban or rural), and administrative regions (Tigray, Afar, Amhara, Oromia, Somali, Benishangul, SNNPR, Gambela, Harari, Diredawa, and Addis Ababa).

### 2.6. Data Analysis Procedures

Data was analyzed using STATA version 14 software. Variables with *P* value <0.05 in bivariate binary logistic regression were included in the multivariate binary logistic regression analysis. Adjusted odds ratio (AOR) with 95% confidence interval (CI) was used to report the association between dependent and independent variables. Statistical significance of the final model was set at *P* value <0.05. The fitness of the model was tested using Hosmer and Lemeshow goodness of fit statistics (*P*=0.71). Multicollinearity between independent variables was checked using variance inflation factor (VIF). The mean value of VIF was 1.95, which is less than the cutoff point [36].

### 2.7. Ethical Considerations

This study used secondary data from the Ethiopian Demographic and Health Survey which was conducted upon the approval of the Ethiopian National Research Ethics Review Committee. The permission to access the datasets was obtained from the MEASURE DHS project website.

## 3. Results

### 3.1. Characteristics of Study Participants

A total of 610 overweight/obese men and 2440 men with normal body mass index were included in this analysis. The age of the study participants ranges from 20 to 59 years old. Five hundred forty-four cases (82.9%) and 702 (28.8%) of controls were urban dwellers. With regard to wealth index, 561 (91.9%) of cases and 1235 (50.6%) of controls were rich. Four hundred forty-four (72.8%) of cases and 1736 (71.1%) of controls were married. In terms of religion, 380 (62.3%) of cases and 1115 (45.7%) of controls were Orthodox (Table 1).

### 3.2. Determinants of Overweight or Obesity

In the final multivariate binary logistic regression analysis, age, marital status, educational level, frequency of watching television, wealth index, place of residence, and region of residence were significantly associated with being overweight or obese. The odds of being overweight or obese were 2.2 times (AOR = 2.2, 95% CI: 1.6–3.0) and 3.4 times (AOR = 3.4, 95% CI: 2.5–4.7) higher among men aged 30–39 years and ≥40 years, respectively, compared to men aged 20–29 years. Married men had 1.5 times (AOR = 1.5, 95% CI: 1.1–2.0) higher odds of being overweight or obese compared to singles. Men with primary, secondary, and higher education had 1.6 times (AOR = 1.6, 95% CI: 1.1–2.3), 2.6 times (AOR = 2.6, 95% CI: 1.7–3.9), and 3.6 (AOR = 3.6, 95% CI: 2.4–5.6) higher odds of being overweight or obese, respectively, compared to men with no education. Men watching television at least once a week had 1.5 times (AOR = 1.5, 95% CI: 1.1–2.1) higher odds of overweight or obesity compared to men not watching television at all. Men living in urban areas were 3.1 times (AOR = 3.1, 95% CI: 2.1–4.4) more likely to be overweight or obese compared to rural. The likelihood of being overweight or obese was higher among men in rich quintiles (AOR = 1.9, 95% CI: 1.2–2.9) compared to men in the poor quintiles. In terms of regional distribution, men living in Addis Ababa (AOR = 1.7, 95% CI: 1.1–2.7), Harari (AOR = 1.8, 95% CI: 1.1–3.2), and Afar (AOR = 2.0, 95% CI: 1.1–3.9) were more likely to be overweight or obese compared to men living in Tigray region (Table 2).

## 4. Discussion

This study used a case-control study to assess determinants of overweight or obesity among men aged 20–59 years in Ethiopia. The study used the 2016 Ethiopian Demographic and Health Survey data, which was a nationally representative survey with an adequate sample size. In this study, age, marital status, educational status, frequency of television watching, wealth index, place of residence, and region of residence were found to be determinants of overweight or obesity.

The likelihood of overweight or obese was 3.1 times higher among urban dwellers compared to rural dwellers. This finding is consistent with studies conducted in Ethiopia [37–39] and elsewhere [38, 40, 41]. This could be related to the fact that urban residents are more likely to follow the Western diet, more likely to use public transportations to go to work daily, and less likely to engage in physically demanding work which might expose them to overweight or obesity [42]. On the contrary, men living in rural areas are more likely to engage in physically demanding work such as agricultural activities and less likely to gain excessive weight.

The odds of being overweight or obese were higher among residents of Addis Ababa and Harari compared to residents of the Tigray region. This might be related to the fact that Addis Ababa and Harari are entirely urban and residents of these areas might be exposed to overweight or obesity as a consequence of lifestyle related to urbanization. This finding is similar to previous studies conducted in Ethiopia [26, 43]. Similarly, residents of the Afar region were more likely to be overweight or obese. These areas were drought-prone areas and more likely to consume food items supplied by humanitarian aid [44] which might expose them to overweight or obesity [45]. This finding is supported by previous studies in Ethiopia [38, 43].

Men from the rich wealth quintile were 1.9 times more likely to be overweight or obese compared to their counterparts. This study also revealed that men with secondary and higher education were more likely to be overweight or obese compared to men with no education. The wealth quintile may be a proxy indicator for characteristics such as access to quality health care, being higher income, dietary diversification, and consumption of energy-dense food. Additionally, educated and rich men are less likely to engage in physically demanding work and more likely to follow a sedentary lifestyle. This finding is supported by multiple studies in Ethiopia and elsewhere [26, 30, 39, 41, 46, 47]. However, some studies reported a negative association between educational status and overweight or obesity [48–50]. It is important to further explore the impact of education on the body mass index with a strong study design.

This analysis indicated that older age and married men had higher odds of being overweight or obese. In line with this study, multiple studies supported a positive association between age and risk of being overweight or obese [26, 30, 37, 40, 41, 46]. As age increases, the level of physical exercise tends to decrease, which increases the risk of being overweight or obese. Likewise, the condition of being overweight or obese was higher among married men compared to unmarried men [40, 51–53]. Married men are less likely to worry about their body image which in turn increases the risk of being overweight or obese. It might also be explained by the fact that a big body size is considered a sign of success and good health, which could contribute to married men being overweight or obese.

Men watching television at least once a weak had 1.8 times higher odds of being overweight or obese compared to those not watching television at all. This finding is corroborated by several studies [54–61]. Men who have a habit of television watching are more likely to stay physically inactive and they are more likely to be exposed to the advertisement of alcohol and unhealthy foods which increases the risk of being overweight or obese.

This study tried to assess the determinants of overweight or obesity among men aged 20–59 years of age. Among the strength of the study, this is the first study to assess the determinants of obesity among Ethiopian men using Demographic and Health Survey data which is nationally representative data. Furthermore, anthropometric measurements were measured by trained data collectors and validated instruments. However, the findings of this study should be used in light of the following limitations. First, due to the cross-sectional nature of the data, the cause-effect relationship between outcome and independent variables cannot be established. Secondly, due to the secondary nature of the data, some important variables which can affect the likelihood overweight or obesity such as dietary habit and physical activity were not measured. Lastly, indicators of central obesity like waist and hip circumferences were not measured by this survey.

## 5. Conclusion

The study assessed the determinants of overweight or obesity. Men with characteristics like higher wealth quintile, older age, higher educational status, and urban dwellers were more likely to be overweight or obese. Being married, watching television at least once a week, and being residents of big cities such as Addis Ababa and Harari, and residents of lowland like Afar were also found to be significant determinants of overweight or obesity. Therefore, it is essential to design strategies and programs to reduce or prevent overweight or obesity with a special focus on identified risk factors in this study and other similar findings.

## Figures and Tables

**Table 1:** Sociodemographic characteristics of men aged 20–59, EDHS, 2016.

Variables	Cases, *n* (%) *N* = 610	Control, *n* (%) *N* = 2440
Age category		
20–29	131 (21.5)	935 (38.3)
30–39	212 (34.7)	735 (30.1)
≥40	267 (43.8)	770 (31.6)
Marital status		
Single	139 (22.8)	613 (25.1)
Married	444 (72.8)	1736 (71.1)
Separated/divorced/widowed	27 (4.4)	91 (3.8)
Religion		
Orthodox	380 (62.3)	1115 (45.7)
Muslim	157 (25.7)	859 (35.2)
Protestant	63 (10.3)	424 (17.4)
Others^a^	10 (1.6)	42 (1.7)
Educational level		
No education	50 (8.2)	792 (32.5)
Primary	134 (22.0)	951 (39.0)
Secondary	165 (27.0)	371 (15.2)
Higher	261 (42.8)	326 (13.3)
Currently working		
No	35 (5.7)	198 (8.1)
Yes	575 (94.3)	2242 (91.9)
Frequency of watching television		
Not at all	82 (13.5)	1171 (48.0)
Less than once a week	95 (15.6)	629 (25.8)
At least once a week	433 (70.9)	640 (26.2)
Frequency of listening radio		
Not at all	142 (23.3)	1079 (44.2)
Less than once a week	135 (22.1)	612 (25.1)
At least once a week	333 (54.6)	749 (30.7)
Frequency of reading newspaper or magazine		
Not at all	236 (38.7)	1676 (68.7)
Less than once a week	193 (31.6)	520 (21.3)
At least once a week	181 (29.7)	244 (10.0)
Wealth index		
Poor	38(6.3)	840 (34.4)
Middle	11 (1.8)	365 (15.0)
Rich	561 (91.9)	1235 (50.6)
Place of residence		
Urban	506 (82.9)	702 (28.8)
Rural	104 (17.1)	1738 (71.2)
Region		
Tigray	37 (6.1)	237 (9.7)
Afar	23 (3.8)	102 (4.2)
Amhara	23 (3.7)	363 (14.9)
Oromia	39 (6.4)	348 (14.3)
Somali	29 (4.8)	143 (5.8)
Benishangul	24 (3.9)	181 (7.4)
SNNPR^b^	39 (6.4)	372 (15.2)
Gambela	31 (5.1)	160 (6.6)
Harari	51 (8.4)	116 (4.7)
Addis Ababa	232 (38.0)	252 (10.3)
Dire dawa	82 (13.4)	166 (6.8)

^a^Catholic, traditional, and others. ^b^South Nation, Nationalities, and People's Region.

**Table 2:** Determinants of overweight or obesity among men aged 20–59, further analysis of EDHS, 2016.

Variables	COR (95% CI)	AOR^a^ (95% CI)
Age category		
20–29	1	1
30–39	2.1 (1.6–2.6)	2.2 (1.6–3.0)^*∗∗*^
≥40	2.5 (1.9–3.1)	3.4 (2.5–4.7)^*∗∗*^
Marital status		
Single	1	1
Married	1.1 (0.9–1.4)	1.5 (1.1–2.0)^*∗∗*^
Separated/divorced/widowed	1.3 (0.8–2.1)	1.0 (0.6–1.8)
Educational level		
No education	1	1
Primary	2.2 (1.6–3.1)	1.6 (1.1–2.3)^*∗∗*^
Secondary	7.0 (5.0–9.9)	2.6 (1.7–3.9)^*∗∗*^
Higher	12.7 (9.1–17.6)	3.6 (2.4–5.6)^*∗∗*^
Currently working		
No	0.7 (0.5–0.9)	0.6 (0.4–1.0)
Yes	1	1
Frequency of watching television		
Not at all	1	1
Less than once a week	2.1 (1.6–2.9)	0.8 (0.5–1.2)
At least once a week	9.6 (7.5–12.5)	1.5 (1.1–2.1)^*∗∗*^
Frequency of listening to the radio		
Not at all	1	1
Less than once a week	1.7 (1.3–2.2)	1.2 (0.8–1.7)
At least once a week	3.4 (2.7–4.2)	1.1 (0.9–1.6)
Frequency of reading newspaper or magazine		
Not at all	1	1
Less than once a week	2.6 (2.1–3.3)	0.9 (0.7–1.2)
At least once a week	5.3 (4.2–6.7)	1.1 (0.8–1.4)
Wealth index		
Poor	1	1
Middle	0.7 (0.3–1.3)	0.6 (0.3–1.3)
Rich	10.0 (7.1–14.1)	1.9 (1.2–2.9)^*∗∗*^
Place of residence		
Urban	12.0 (9.6–15.1)	3.1 (2.1–4.4)^*∗∗*^
Rural	1	1
Region		
Tigray	1	1
Afar	1.4 (0.8–2.6)	2.0 (1.1–3.9) ^*∗∗*^
Amhara	0.4 (0.2–0.7)	0.6 (0.3–1.1)
Oromia	0.7 (0.4–1.1)	1.0 (0.6–1.8)
Somali	1.3 (0.8–2.2)	1.5 (0.8–2.8)
Benishangul	0.8 (0.5–1.5)	1.4 (0.7–2.6)
SNNPR^b^	0.7 (0.4–1.1)	1.0 (0.6–1.9)
Gambela	1.2 (0.7–2.0)	0.9 (0.5–1.6)
Harari	2.8 (1.7–4.5)	1.8 (1.1–3.2)^*∗∗*^
Addis Ababa	5.9 (4.0–8.7)	1.7 (1.1–2.7)^*∗∗*^
Dire dawa	3.2 (2.0–4.9)	1.4 (0.9–2.4)

^a^Adjusted for age, marital status, educational status, television watching, residence, wealth index, and region. ^b^South nation, nationalities, and people's region. ^*∗∗*^Statistically significant in multivariate logistic regression model at *p* value <0.05.

## Data Availability

The data that support the findings of this study are available from the MEASURE DHS project upon reasonable request after submission of the concept paper.

## Abbreviations

AOR: Adjusted odds ratio
CI: Confidence interval
COR: Crude odds ratio
DHS: Demographic and Health Survey
EDHS: Ethiopian Demographic and Health Survey.
